# Comparison of analgesic effect of preoperative topical Diclofenac versus Ketorolac on postoperative pain after Corneal Collagen Cross Linkage

**DOI:** 10.12669/pjms.335.13247

**Published:** 2017

**Authors:** Murtaza Sameen, Muhammad Saim Khan, Asad Habib, Muhammad Amer Yaqub, Mazhar Ishaq

**Affiliations:** 1 Dr. Murtaza Sameen, MBBS. Armed Forces Institute of Ophthalmology, Rawalpindi, Pakistan; 2 Dr. Muhammad Saim Khan, FCPS, FICO, MRCSEd. Armed Forces Institute of Ophthalmology, Rawalpindi, Pakistan; 3 Dr. Asad Habib, MBBS. Armed Forces Institute of Ophthalmology, Rawalpindi, Pakistan; 4 Dr. Muhammad Amer Yaqub, MCPS, FCPS, FRCSEd. Armed Forces Institute of Ophthalmology, Rawalpindi, Pakistan; 5 Prof. Dr. Mazhar Ishaq, FCPS, FRCSEd, FRCOphth. Armed Forces Institute of Ophthalmology, Rawalpindi, Pakistan

**Keywords:** Corneal Crosslinking, Diclofenac, Keratoconus, Ketorolac, Pain

## Abstract

**Objective:**

To compare post-operative pain relieving effect of topical diclofenac 0.1% versus ketorolac 0.5% in Corneal Collagen Cross Linking (CXL) for patients diagnosed with keratoconus.

**Methods:**

This randomized controlled trial was carried out for six months from October 2016 to March 2017. We included young patients having keratoconus with k-readings greater than 47D and central corneal thickness more than 400 microns. All the patients received single dose one drop of topical diclofenac 0.1% to (Group-A) and ketorolac 0.5% to (Group-B) 30 minutes in advance of the corneal collagen cross linking (CXL) procedure. The CXL was performed with topical 0.1% riboflavin eye drops in 20% dextran as a photo sensitizer. After 36 hours of the CXL procedure, the postoperative intensity of pain was assessed verbally by patients with the help of visual analog scale (VAS) numbers from zero to five where 0 designated no pain & 5 symbolized worst pain.

**Results:**

The study comprised sixty eyes of forty one patients. Out of total 16 were male while 25 female patients. The mean age of the patients was 24.27 ± 2.93 years (range 20 to 29 years). In the conclusive analysis, diclofenac 0.1% was used on 30 patients in Group-A and ketorolac 0.5% on 30 subjects in Group-B. Pain relieving scores in Group-A (diclofenac 0.1%) was 2.57 ± 0.67 while in Group-B (ketorolac 0.4% treated arm) was 3.20 ± 0.61.

**Conclusion:**

Topical diclofenac 0.1% is statistically comparable to topical ketorolac 0.5% in precluding severity of pain after corneal collagen cross linkage operation.

## INTRODUCTION

Keratoconus is a non-inflammatory progressive disease characterized by conical cornea inducing myopic irregular astigmatism. Keratoconus affects both the sexes equally and its onset of generally occurs at puberty.^1^ The global incidence of keratoconus in general population is estimated to be 1 in 2000.^2^ Corneal collagen cross-linkage (CXL) was introduced by Eberhard Spoesi and Theo Seiler in 1990 as treatment of keratoconus.^3^ This is a quite reputed method of refractive surgical intervention which crops cross linking of collagen using Riboflavin (Vit-B2) and ultraviolet-A (UVA) irradiation. The UV-A ultimately acts as photo sensitizer resulting in development of free radicals. The outcome is triggering of natural lysl oxidase pathway, that effects immediately after irradiation leading to an increase of the biomechanical rigidity of the cornea of about 300%.^4^ Recent management protocol entails use of UV-A energies of 3 mW/cm^2^ for about 30 min to gain clinical benefits.^4^

Customary CXL-procedure familiarized by Wollensak and colleagues recommended a short duration refractive surgical technique comprised of mechanical peeling of corneal epithelium around 7mm diameter with use of 0.1% isotonic riboflavin solution in 20% dextran as a photosensitizer.^5^ Many studies have publicized that CXL can often avert the need for a corneal transplant and enable patients to wear contact lenses or glasses more restfully and securely again. CXL, by persuading corneal flattening is efficacious in slackening or stumbling headway of keratoconus and may even validate visual and topographic corneal improvement thus resulting into cutback in irregular astigmatism.^6^ Postoperative ocular inflammation is an indigenous comeback that instigates proximately subsequent to surgical trauma. Modest to severe pain is reported to be the significant menace, particularly during the first postoperative 24 hours succeeding CXL. This is usually due to ablation of corneal surface epithelium resulting in exposure of highly sensitive nerves and the consequent inflammation.^7^,^8^

Non-steroidal anti-inflammatory drugs (NSAIDs) are quite well known to exercise potent anti-inflammatory and analgesic effects. There is evidence that non-steroidal anti-inflammatory (NSAIDs) preparations are administered topically before refractive surgery as they lower down post-surgical intensity of pain and photophobia.^9^-^11^ Multiple studies have revealed the influence of diclofenac and ketorolac in fading pain along with corneal sensitivity consequent to corneal abrasion.^4^ They have a little action on the Cyclooxygenase (COX) enzyme activity and necessitate deamination to the more active congener amfenac in order to promote abrupt therapeutic action. In the United States, Ketorolac & diclofenac are the NSAIDs of choice in refractive surgery.^9^ The aim is to control pain for more than 36 hours as the onset of maximum pain is within first 24 and 36 hours. Instillation of one drop of Diclofenac 0.1% and ketorolac 0.4% and 0.5%, 30 minutes prior to start the CXL procedure is the recommended dosage to minimize post-operative pain.^12^

Armed Forces Institute of Ophthalmology is a tertiary care center with well-equipped and state of the art refractive surgery department. To best of our knowledge in the field of refractive surgery, no study has yet been conducted at the national level to differentiate topical ketorolac with topical diclofenac in terms of post CXL pain relief. Therefore, we designed this study to compare the usefulness of a single dose of topical diclofenac 0.1 % and ketorolac 0.5 % with each other in the preclusion of post-CXL pain and discomfort.

## METHODS

This was a randomized control clinical study conducted at the Armed forces institute of ophthalmology (AFIO) Rawalpindi between October 2016 and March 2017. This single-station and single surgeon prospective randomized study comprised of male and female patients between 16 to 35 years of age, included by non-probability consecutive sampling suffering from significant keratoconus with k-readings greater than 47D and central corneal thickness more than 400um. The subjects with any previous history of ocular surgery, acute corneal hydrops, dense corneal ectasia, corneal opacity, any history of hypersensitivity against the NSAIDs or use of topical/systemic anti-inflammatory medications within two weeks before surgery were excluded from the study. The study protocol was approved by the Ethics/Research Committee of Armed Forces Institute of Ophthalmology (AFIO) Rawalpindi. All registered subjects signed a written informed consent. A total of 60 eyes of 41 patients were investigated in this study. All eyes that underwent CXL between October 2016 and March 2017 were scrutinized. The pre-operative assessment included un-corrected visual acuity (UCVA), best spectacle-corrected visual acuity (BCVA) and detailed examination of anterior and posterior segments with slit lamp. Investigations such as corneal topography (using Galilei-G4) and ultrasound pachymetry were performed. The enrolled patients were randomly divided into two groups and assigned to two arms of drug regimen. Group-A to Diclofenac 0.1% and Group-B to Ketorolac 0.5%.

### Procedure

The first arm Group-A received one drop of diclofenac sodium 0.1%, and the second arm Group-B received one drop of ketorolac 0.5% thirty minutes before surgical intervention by a qualified and trained coordinator. Under sterile conditions CXL was performed as a day care procedure. Lids and skin scrub was done with 5% povidone-iodine solution. Topical akinesia was induced by using proparacaine hydrochloride 0.5% ophthalmic drops. A central 7 mm round corneal deepithelialization for efficient penetration of riboflavin through exposed corneal stroma was performed with the help of sterile blunt spatula. Riboflavin 0.1% in 20% dextran was administered topically every two minutes for 30 minutes. Slit lamp examination was performed to confirm riboflavin absorption throughout the corneal stroma and anterior chamber. Riboflavin saturation in corneal stroma ensures the formation of free radicals whereas riboflavin shielding in anterior chamber sub serve protection of deeper ocular structures such as the corneal endothelium. UVA radiations were delivered using (CCL-365) corneal cross-linking system at a distance of 5-6 cm from cornea for 30 minutes at power settings of 3mW/cm2 (Standard Dresden Protocol). Bandage soft contact lens was placed at the end of procedure for three days till complete reepithelialization of cornea. All the operated patients were informed of how to evaluate their post-operative ocular discomfort and subjectively report to the concerned. After 36 hours of CXL procedure, the postoperative intensity of pain was assessed verbally by patients with the help of verbal rating scale (VRS) numbers from zero to five where 0 designated no pain & 5 symbolized worst pain. In a much selected patients with post-operative worst pain oral analgesics were advised.

### Statistical Analysis

Statistical Package for Social Sciences (SPSS) version 16 was used for data analysis. Descriptive statistics were used to calculate mean and standard deviation for quantitative. Variables like age, pain. Frequencies & percentages were presented for qualitative variable like gender. To measure difference of mean pain between two groups (Group-A = Diclofenac and Group-B = Ketorolac), independent sample t-test was utilized-value ≤ 0.05 was considered as a significant measure.

## RESULTS

**Fig.1:**
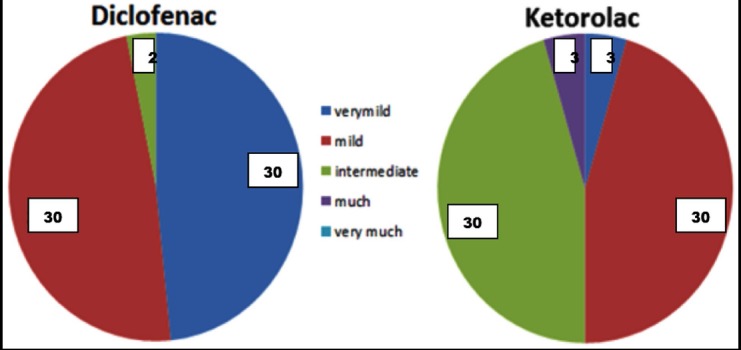
Comparison (Percentage) of various pain severities in Diclofenac versus Ketorolac groups.

Sixty eyes were included in this study, divided into two groups of thirty each. About 66.7% were right eyes and 60% of the participants of study were females. Mean age was 24.27 ± 2.93 years. Both the groups were comparable in terms of gender and age. Total of 33 eyes experienced very mild grade pain (30 in diclofenac group vs. 3 in ketorolac group) after a mean duration of 5.00 ± 1.4 hours after surgery (5.23 ± 0.89 and 2.67 ± 0.57 for Group 1 and 2 respectively, p < 0.05) Fig.1

In all the sixty eyes, pain was graded as **mild** after a mean duration of 5.32 ± 2.22 hours (7.20 ± 1.06 and 3.43 ± 1.25 for Group 1 and 2 respectively, p<0.05) Table-I.

**Table-I:** Mean and Standard deviation of pain free duration of postoperative pain.

*Variables*	*N*	*Min*	*Max*	*Mean + SD*
Age	60	20	29	24.27 ± 2.93
No pain	0	-	-	
Very Mild	33	2	7	5.00 ± 1.14
Mild	60	2	9	5.32 ± 2.22
Intermediate	32	4	9	5.94 ± 1.41
Much	3	6	7	6.33 ±0.57
Very Much	0	-	-	-

Only in two patients pain progressed to **intermediate grade**, after mean duration of 6.50 ± 0.70 hours post op in Diclofenac group versus 30 eyes progressing to intermediate pain in ketorolac group earlier than diclofenac group (5.90 ± 1.44 hours). None of the patient in diclofenac group progressed to severe degree of pain while 3 patients reported it after a mean of 6.33±0.57 hours in ketorolac group. Table-II.

**Table-II:** Mean duration of pain free interval in two treatment groups.

*Groups*	*N*	*Mean ±SD*	*Sig*
Pain Duration Group – 1 (Diclofenac)	30	5.23 ±0.898	P = 0.06
Pain Duration Group – 2 (Ketorolac)	30	3.43 ± 1.25	
Pain Score Group – 1 (Diclofenac)	30	2.57 ±0.67	P = 0.14
Pain Score Group – 2 (Ketorolac)	30	3.20 ± 0.61	

## DISCUSSION

The only proven treatment that halts progression of Keratoconus is Corneal crosslinking (CXL).^13^,^14^ Apart from other benefits, CXL sometimes delays or even does away with the need of corneal transplantation in patients with keratoconus.^15^ This is a proven fact that the non-steroidal anti-inflammatory drugs (NSAIDs)used topically before CXL trim down post-operative pain and discomfort.^16^

El-Harazi SM and Kim SJ in their studies also observed significant regression in post-operative anti-inflammatory effect in subjects using preemptive NSAIDs.^10^,^17^

There are many techniques and methods for management of postoperative pain; of these, pre-operative approach in the use of NSAIDs for inducing analgesia is of great interest. Presumptive use of NSAIDs is quite efficacious in the first post-operative day to control pain and discomfort.^18^ In our study, we found out that pain of various intensity observed in all 60 eyes of the patients; however its intensity was different in the two groups. Patients in Group-1, who were pretreated with diclofenac 0.1% had lower mean pain score (2.57 ± 0.67) in the early phase of study i.e.36 hours post-operatively than the eyes that received ketorolac tromethamine 0.5% (3.20 ± 0.61). Unlike study conducted by Mohammadpour M et al. who concluded that diclofenac sodium 0.01% ophthalmic suspension was more effective, safe, and well tolerated in the post-operative management of pain, our study revealed different results.^18^ We found that the difference of pain score between two groups is statistically insignificant (P=0.06).^18^

In this study, it was also observed that pain free interval after CXL was longer in diclofenac group (7.20+1.06 hours) as compare to ketorolac group (3.43+1.25 hours). However, this pain relief interval is statistically insignificant (P = 0.14)and the duration is much less than what has been claimed by Hang JP et al, who claimed this to be 24 hours.

Maurin et al. studied pain perception in Epi-off versus Epi-on CXL using iontophoresis and found out statistically less pain in later as compared with earlier. Ghanem et al studied relation of age and pain perception and found out a statistically significant difference between pain perception between the two age groups.^19^,^20^ Younger patients tend to perceive more pain after CXL compared to old patients. However these variables were not studied in our study. All the participants in our study were young with a mean age of 24.27 + 2.93 years and all CXL were Epi-off.

### Limitations of the Study

It has small sample size and short-term follow-up. The short term and long term snags of CXL like, temporary corneal haze, corneal infection, and prolonged post-CXL ocular pain and discomfort were not considered. Further additional work is required for pre-operative analgesia in ophthalmic surgical procedures to overcome early and prolonged post CXL pain related hazards in the management of progressive keratoconus.

## CONCLUSION

We concluded that topical diclofenac 0.1% is comparable with topical ketorolac 0.5% in releieving postoperative pain following CXL surgery.

### Authors` Contribution

**MS:** Conception and Data collection.

**MSK:** Designed and drafting the manuscript. **AH:** Conception and Design. **MAY:** Final review. **MI:** Final approval of the manuscript.
